# Soil Nutrients, pH and Microorganisms Modulate Nitrogen Mineralization Dynamics Following Afforestation in Northeastern China

**DOI:** 10.3390/plants15121892

**Published:** 2026-06-18

**Authors:** Lei Guo, Xu Cao, Ruihan Xiao, Kexin Tong, Tao Liu, Minghan Lang, Beixing Duan

**Affiliations:** 1 School of Hydraulic and Electric Power, Heilongjiang University, Harbin 150080, China; guolei@s.hlju.edu.cn (L.G.); coxi@s.hlju.edu.cn (X.C.); xiaoruihan@hlju.edu.cn (R.X.); 2022074@hlju.edu.cn (K.T.); 2002219@hlju.edu.cn (T.L.); 2 International Joint Laboratory of Hydrology and Hydraulic Engineering in Cold Regions of Heilongjiang Province, Harbin 150080, China; 3 Post-Doctoral Mobile Research Station of Ecology, Heilongjiang University, Harbin 150080, China; 4 Liaoning Zhanggutai Desert Ecosystem Research Station, Liaoning Institute of Sandy Land Control and Utilization, Fuxin 123000, China; 18804502009@163.com

**Keywords:** afforestation, Alfisols, soil nitrogen mineralization, soil nutrients, soil microbes, larch forest

## Abstract

Grain for Green, as an important ecological restoration method, profoundly affects soil nitrogen (N) cycling by altering the soil physicochemical properties and microbial community. Soil nitrogen mineralization is a key process in the terrestrial N cycle. However, the dynamics and underlying driving mechanisms of soil N mineralization rate (Rmin) that respond to afforestation remain unclear. In this study, we selected a typical afforestation sequence in Northeast China, including farmland (F), 21-year-old larch plantation (L21), 42-year-old larch plantation (L42), and natural larch forest (NL). The soil Rmin, associated soil physicochemical properties, and microbial community characteristics were determined to explore the effects of afforestation on soil Rmin and its potential mechanisms of action. The results suggested that soil Rmin was ranked in the order of L42 (0.41 mg kg^−1^ d^−1^) > F (0.39 mg kg^−1^ d^−1^) > L21 (0.23 mg kg^−1^ d^−1^) (*p* < 0.05) along the afforestation sequence, with no significant difference between L42 and F. Compared to the L42, the NL exhibited significantly lower soil Rmin (0.23 mg kg^−1^ d^−1^) (*p* < 0.05). The changes in soil Rmin during the afforestation were significantly positively related to soil total N (TN) and organic carbon (SOC) concentrations, but significantly negatively related to pH (*p* < 0.05). Furthermore, the abundances of *Proteobacteria* and *Acidobacteria* (bacteria) and *Ascomycota* (fungi) were also closely correlated with soil Rmin. Structural equation modeling (SEM) analysis further indicated that the afforestation mainly regulated soil Rmin by altering soil temperature (ST) and NH_4_^+^-N content. Meanwhile, soil NH_4_^+^-N content could also exert a significantly positive effect on soil Rmin by influencing the microbial community. In conclusion, afforestation effectively altered soil Rmin, which was even higher in the plantation than in natural forests. This finding further enhances our understanding of forest restoration and land management practices on soil N cycling in temperate regions.

## 1. Introduction

Forests in Northeast China act as important ecological barriers for Northeast and even North China and play crucial roles in regulating regional and global climate change [1]. However, in past decades, deforestation has expanded rapidly across most Northeast regions due to socioeconomic pressures, and the consequent large-scale conversion of natural forests to farmland has resulted in the structural fragmentation and functional degradation of ecosystems [2]. Deforestation has not only caused a loss of vegetation cover but has also disrupted the soil N cycle, consequently severely reducing soil N supply capacity [3]. Therefore, it has become an urgent issue to reverse the trend of ecological degradation and restore forest ecosystem functions in Northeast China. As the largest ecological restoration program in China, the Grain-for-Green project (GGP) has led to a large amount of land-use change and profoundly affected soil ecological processes, especially for N transformation [4]. Soil nitrogen mineralization is the key process in which soil organic N can be transformed to inorganic N, which plays a vital role in determining nutrient availability and ecosystem productivity [5]. Therefore, it is essential to reveal the dynamics and regulatory mechanisms of the soil N mineralization rate (Rmin) during afforestation in Northeast China, which can provide critical evidence for scientifically evaluating the ecological benefits of GGP in restoring soil nutrient supply functions.

Numerous studies have attempted to investigate the influences of afforestation on Rmin; however, the results are not consistent. Previous studies have reported a significant increase [6], decrease [7], and no significant changes [8,9] in soil Rmin following the afforestation. These differences could be due to the fact that soil Rmin is regulated by multiple factors, such as the microenvironment, soil physicochemical properties, and microorganisms. Additionally, the afforestation years could also have significant effects on soil Rmin. Previous studies have revealed that soil Rmin increases significantly with afforestation years [10]. However, Rong et al. [11] suggested that Rmin continuously increased only during the first 30 years following afforestation, after which a marked decrease occurred. In contrast, Li et al. [12] demonstrated that Rmin significantly increased with afforestation initially, showing no significant differences across 10 to 30 afforestation years. In recent decades, large-scale afforestation efforts under GGP have been extensively implemented across Northeast China in response to ecological restoration imperatives. Nevertheless, the long-term effects and regulatory mechanisms of afforestation on soil Rmin in this region remain poorly characterized. This knowledge gap may limit our understanding of the recovery of soil N cycling and nutrient function following afforestation.

Afforestation is usually accompanied by changes in soil microenvironment, pH, soil water content (SWC), and substrate availability [11]. Although these changes could individually/interactively affect the soil Rmin, the predominant factors are significantly different. For instance, Hu et al. [13] found that Rmin was mainly regulated by pH and soil organic matter, and was negatively correlated with pH. However, studies in the Karst area have suggested a close positive correlation between soil Rmin and pH, which is mainly due to the fact that an increase in soil pH can enhance substrate availability and ultimately promote soil Rmin [14]. Elrys et al. [5] found that higher SWC indirectly promotes gross nitrogen mineralization by raising total nitrogen content and reducing soil bulk density. In contrast, drought stress elevates fungal proportion in microbial communities and suppresses gross nitrogen mineralization. Meanwhile, the important roles of organic carbon (SOC), total nitrogen (TN), and the C:N ratio in regulating soil Rmin have also been reported [15,16]. Given that the different patterns of these factors along afforestation, it is still unresolved which factors dominate in regulating soil Rmin.

Meanwhile, soil microbes also play fundamental roles in the soil N cycle, and their community structure, diversity, and abundance significantly affect soil Rmin. Previous studies have shown that soil Rmin is significantly correlated with soil microbial community structure [10]. Most studies hold that bacteria are the primary actors in regulating Rmin, which exerts its role by decomposing organic N and mediating ammonification and nitrification [17,18,19]. Specifically, changes in the abundance of *Proteobacteria*, *Actinobacteria*, *Acidobacteria*, and *Nitrospirae* significantly affect soil Rmin [5,18,20,21]. However, other studies have also found a tighter coupling of fungal communities and soil Rmin [22]. Alterations in the abundance of *Ascomycota* are directly linked to the soil Rmin [23]. Additionally, soil Rmin is also closely related to the variations in microbial biomass [24,25]. However, there are significant differences in patterns of soil microbial diversity, community structure, and abundance following afforestation among different studies [9,26,27,28]. Thus, the underlying pathways and mechanisms of the effects of afforestation on soil Rmin remain to be further revealed.

The GGP was widely implemented in 2003 in Northeast China. More than 18.53 million hectares of farmland have been transformed into forests. However, Northeast China is characterized by a lower annual temperature, which may affect the soil Rmin during afforestation. In this study, a typical afforestation sequence, including farmland (F), 21-year-old larch plantation (L21), 42-year-old larch plantation (L42), and natural larch forest (NL), was selected in Northeast China. The objectives of our study were to (i) explore the spatiotemporal trends in soil Rmin and its influencing factors with the afforestation, and (ii) elucidate the regulatory pathways of afforestation on soil Rmin by combining the changes in soil physicochemical properties and microbial communities. We hypothesized that (1) Soil Rmin and nutrient availability would increase with afforestation. (2) This increase may lead the Rmin in the plantations to approach the level of that in NL over time. (3) Variations in soil microbial communities likely accounted for the changes in soil Rmin along with the afforestation.

## 2. Results

### 2.1. Soil Inorganic N

**Figure 1:**
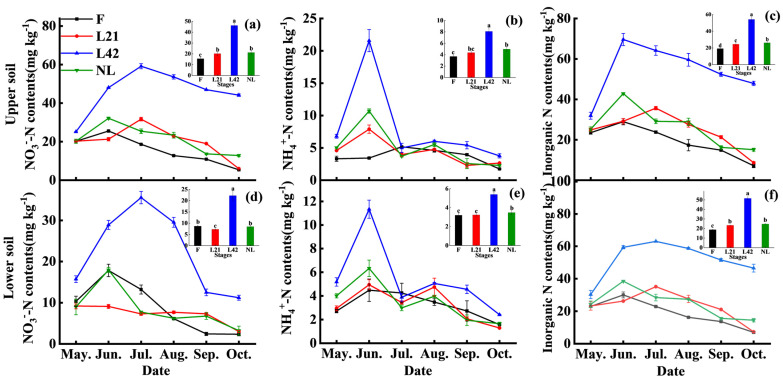
Seasonality dynamics of the contents of soil NO_3_^−^-N (**a**,**d**), NH_4_^+^-N (**b**,**e**), and total inorganic N contents (**c**,**f**) in 0–10 cm and 10–20 cm soil layers with afforestation. Notes: Subplots show the average soil NO_3_^−^-N, NH_4_^+^-N, and inorganic N contents over the study period for each afforestation stage. F, farmland; L21, 21-year larch forest; L42, 42-year larch forest; NL, Natural larch forest. Lowercase letters in subplots indicate significant differences among the different stages.

During the study period, afforestation significantly affected soil inorganic N content (Figure 1). There were significantly higher soil NH_4_^+^-N, NO_3_^−^-N, and total inorganic N contents in L42 than in F, L21, and NL in both soil layers (*p* < 0.05). Soil NH_4_^+^-N, NO_3_^−^-N, and inorganic N contents all showed significant seasonal variations across different afforestation stages, with the highest values occurring in June and July, consistent with growing-season N demand and microbial activity peaks. All inorganic N fractions were significantly higher in the 0–10 cm layer than in the 10–20 cm layer at four stages following the afforestation (*p* < 0.05).

### 2.2. Soil Rnit, Ramm, and Rmin

**Figure 2:**
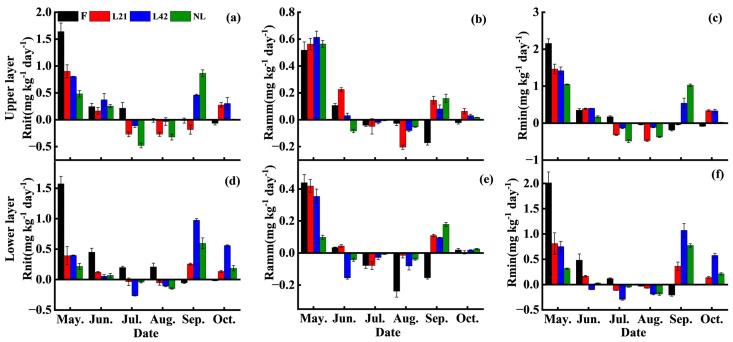
Seasonality dynamics of soil Rmin, Rnit, and Ramm at four afforestation stages in both 0–10 cm and 10–20 cm soil layers. Rnit (**a**,**d**); Ramm (**b**,**e**); Rmin (**c**,**f**). F, farmland; L21, 21-year larch forest; L42, 42-year larch forest; NL, Natural larch forest.

**Figure 3:**
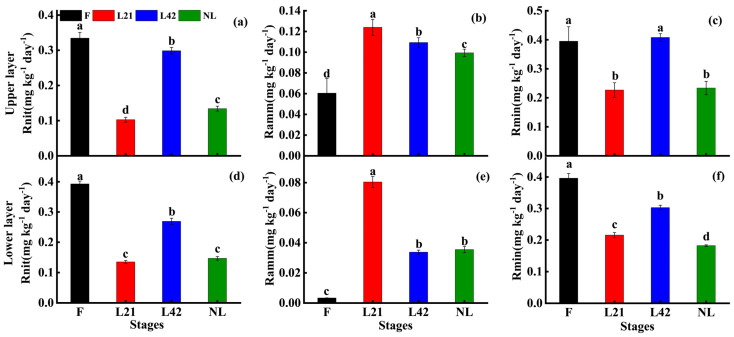
Variations in the average soil Rmin, Rnit, and Ramm in the 0–10 cm and 10–20 cm soil layers following afforestation. Rnit (**a**,**d**); Ramm (**b**,**e**); Rmin (**c**,**f**). F, farmland; L21, 21-year larch forest; L42, 42-year larch forest; NL, Natural larch forest. Lowercase letters indicate significant differences among the different stages.

The afforestation significantly altered the Rnit, Ramm, and Rmin in both soil layers (Figure 2). In our study, the average soil Rnit and Rmin were significantly higher in F and L42 than those in L21 and NL, while Soil Ramm followed the order of L21 > L42 > NL > F in both soil layers (*p* < 0.01; Figure 3). Soil Rnit, Ramm, and Rmin in both soil layers exhibited distinct V-shaped seasonal dynamics at the four stages and remained significantly higher in the 0–10 cm soil layer relative to the 10–20 cm soil layer during the observation period.

### 2.3. Soil Microbial Biomass

**Figure 4:**
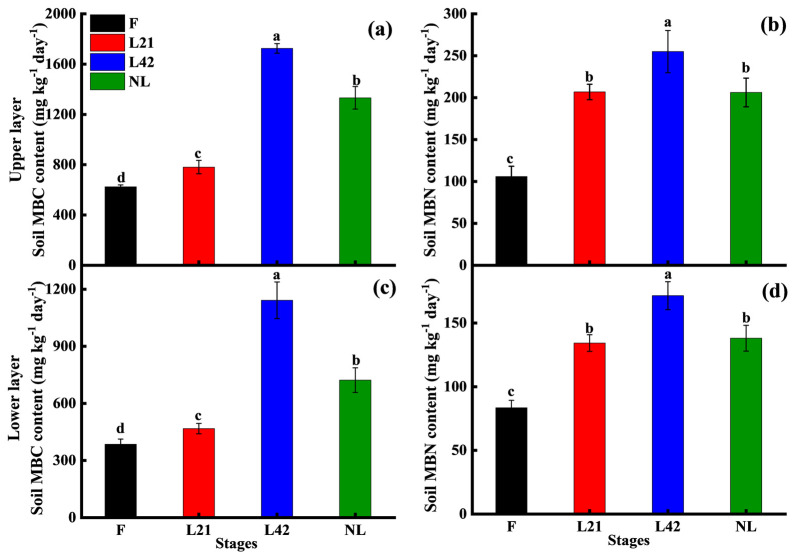
Dynamics of the average contents of soil MBC (**a**,**c**) and MBN (**b**,**d**) in 0–10 cm and 10–20 cm soil layers following afforestation. Notes: Lowercase letters indicate the significant differences among different stages following afforestation. F, farmland; L21, 21-year larch forest; L42, 42-year larch forest; NL, Natural larch forest. Lowercase letters indicate significant differences among the different stages.

**Figure 5:**
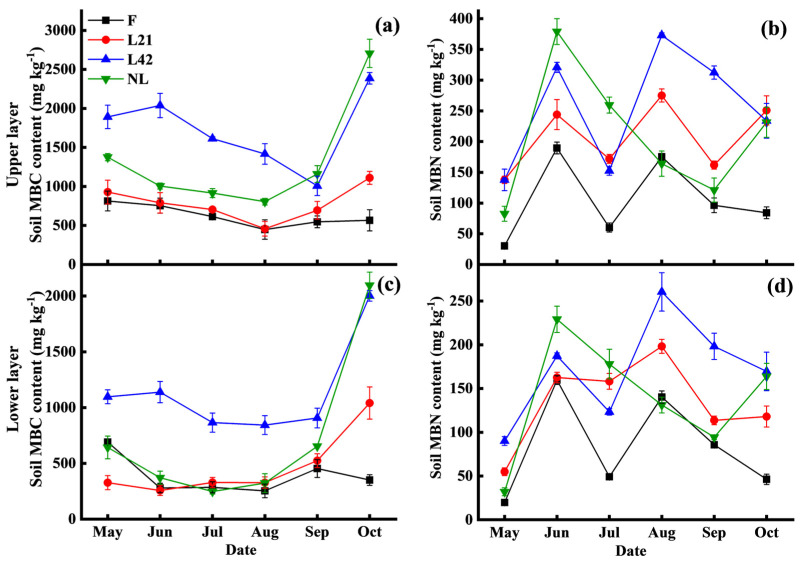
Seasonality dynamics of the contents of soil MBC (**a**,**c**) and MBN (**b**,**d**) at the four stages in 0–10 cm and 10–20 cm soil layers. F, farmland; L21, 21-year larch forest; L42, 42-year larch forest; NL, Natural larch forest.

Soil MBC and MBN contents showed significant differences with afforestation and seasonality in our study (Figure 4 and Figure 5). Average soil MBC and MBN concentrations ranked as L42 > NL > L21 > F (*p* < 0.01) during the study period. However, no significant difference was observed in MBN between L21 and NL (Figure 4). Soil MBC contents in both soil layers across four stages and both soil layers initially decreased and then increased across the study period, with the lowest values occurring in August and September. In contrast, soil MBN in both soil layers at the four stages showed a fluctuating seasonality trend (Figure 5).

### 2.4. Soil Microbial Community Characteristics

**Figure 6:**
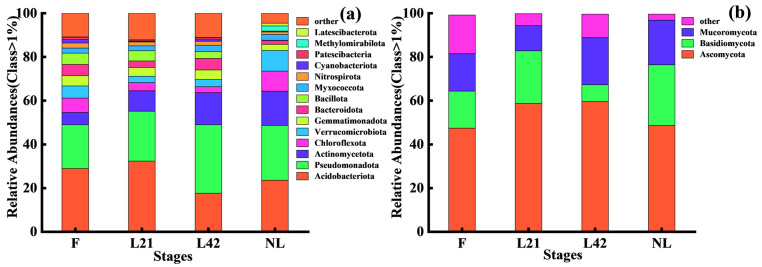
Variations in the relative abundances of soil microbial communities at the phylum level following afforestation. The groups with relative abundances higher than 1% are shown, while those with less than 1% relative abundance are integrated into “other”. Notes: Bacterial community (**a**); Fungal community (**b**). F, farmland; L21, 21-year larch forest; L42, 42-year larch forest; NL, Natural larch forest.

The *Pseudomonadota*, *Actinobacteria*, *Acidobacteria*, *Chloroflexota*, and *Verrucomicrobiota* were the dominant soil bacterial communities at the phylum level among all afforestation stages (Figure 6a). The abundances of *Pseudomonadota* and *Actinobacteria* increased along afforestation, and were significantly higher in L42 than in NL (*p* < 0.05). However, the abundances of *Acidobacteria*, *Chloroflexota*, and *Verrucomicrobiota* decreased with afforestation, which were significantly lower in L42 than in NL (*p* < 0.05). The soil fungal community across the four stages was dominated by *Ascomycota*, *Basidiomycota*, and *Mucoromycota* (Figure 6b). The abundance of *Ascomycota* increased with afforestation, which was lowest in NL than in other stages (*p* < 0.05). However, the abundances of *Basidiomycota* and *Mucoromycota* decreased during afforestation, and were the highest in NL than in other stages (*p* < 0.05).

Soil bacterial and fungal alpha diversities during afforestation are presented in Table 1. The bacterial Chao1 and ACE indices in F, L21, and L42 were significantly lower than those in NL (*p* < 0.05). No significant differences in bacterial Shannon and Simpson indices were found among the four stages. The fungal Chao1 and ACE indices exhibited a decreasing trend with afforestation, and were the lowest in NL (*p* < 0.05). However, the fungal Shannon and Simpson indices in NL were highest among all four stages. (*p* < 0.05).

**Figure 7:**
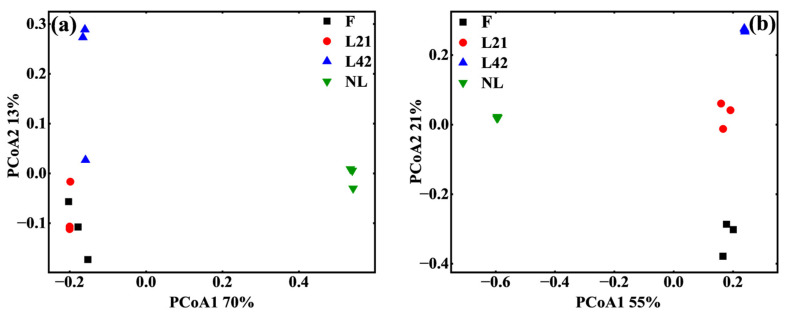
(**a**,**b**) Non-metric multidimensional scaling (PCOA) 2D sorting diagram of the sample plots based on weighted Bray–Curtis and Hellinger distances. Notes: F, farmland; L21, 21-year larch forest; L42, 42-year larch forest; NL, Natural larch forest.

The soil bacterial and fungal community compositions were clearly clustered across the four stages (Figure 7). The PCoA results revealed that afforestation explained 83% and 76% of the variation in soil bacterial and fungal communities, respectively. Notably, obvious differences were observed between NL and the other three afforestation stages in soil bacterial and fungal communities (Figure 7a,b).

### 2.5. Relationships Among Soil Rmin, Soil Microbial Communities, and Properties

**Figure 8:**
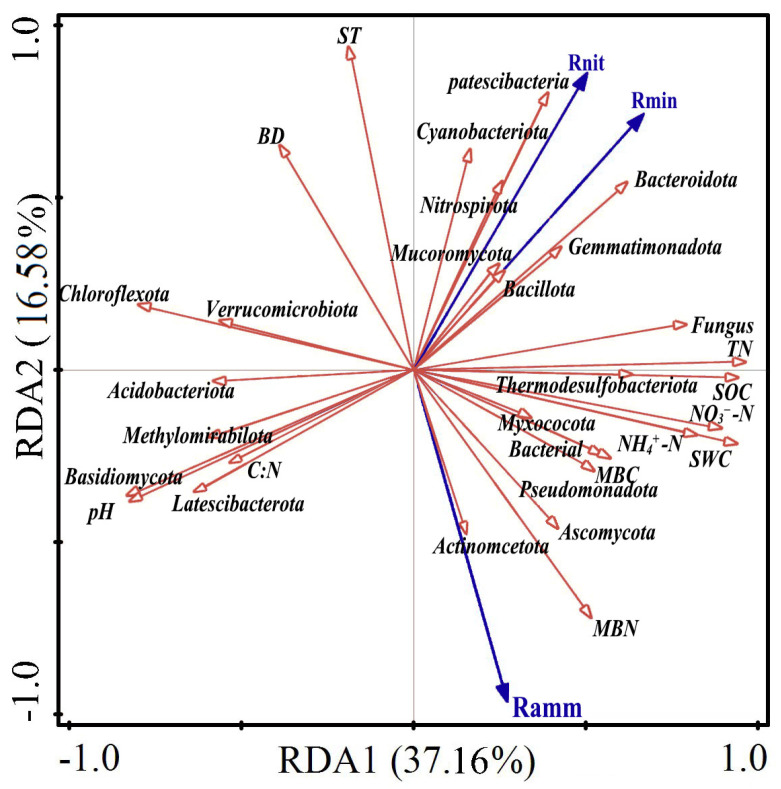
Redundancy analysis bi-plot showing profiles of soil Rmin (including Rnit and Ramm) related to soil edaphic factors and microbial communities. Notes: ST, soil temperature; SWC, soil water content; BD, soil bulk density; pH, soil pH; SOC, soil organic carbon; TN, total nitrogen; MBC, microbial biomass carbon; MBN, microbial biomass nitrogen.

The RDA indicated that soil Rmin was closely correlated with soil properties and microbial communities (Figure 8). The first and second axes explained 37.44% and 16.9% soil Rmin variations, respectively. Soil Rmin was positively correlated with soil fungal abundance. Significant negative correlations were found between both Rnit and Rmin and soil pH (*p* < 0.05). Soil Ramm also showed significant negative correlations with soil BD and ST (*p* < 0.05).

**Figure 9:**
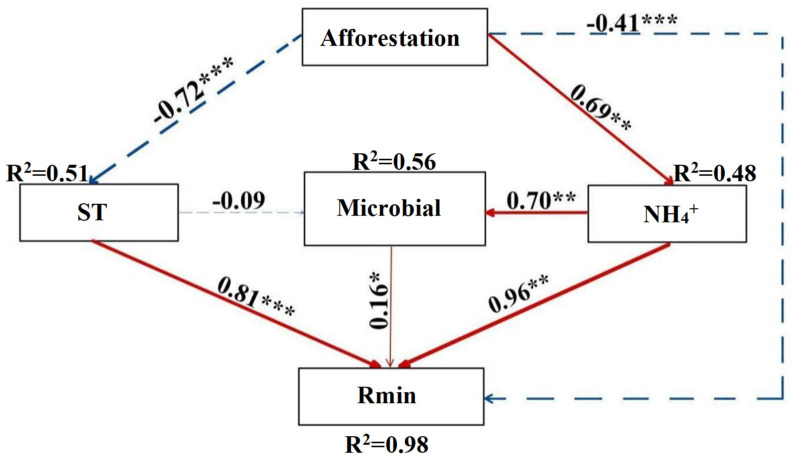
SEM depicting the influence of Grain for Green on soil microbial community and soil Rmin. Notes: Results of model fitting: χ^2^ = 1.005, *p* = 0.729, df = 2; GFI = 1.000; RMSEA = 0.000; AIC = 26.632. Red solid arrows indicate positive effects; blue dotted arrows represent negative effects. R^2^ values represent the proportion of variance explained for each variable. The values beside the arrow represent standardized coefficient. Significance of the correlation is indicated at the 0.001 (***), 0.01 (**), and 0.05 (*) level.

The SEM supported the key pathways through which afforestation affected soil Rmin (Figure 9) and explained 98% of the variance in Rmin. Specifically, afforestation could regulate Rmin directly and indirectly through its effects on soil ST and NH_4_^+^. Soil NH_4_^+^-N content could also affect Rmin directly and exert indirect effects through regulating the soil microbial community. ST also had direct effects on Rmin.

## 3. Discussion

### 3.1. Effects of Afforestation on Rmin

In this study, soil Rmin differed significantly across afforestation stages (Figure 2). Soil Rmin initially decreased and then increased with afforestation years (L42 > F > L21), contrary to our first hypothesis. This trend could be explained by the changes in ST during afforestation. Higher ST could stimulate microbial activity, which subsequently enhances the soil Rmin [29]. In our study, ST initially decreased and then increased (Figure S1). Meanwhile, studies have demonstrated that afforestation directly regulates soil N transformation by altering the organic matter inputs to the soil [30]. In this study, the soil organic carbon (SOC) in L42 was significantly higher than in F and L21, resulting in the highest Rmin in L42. Although there was no significant difference in SOC between F and L21, Rmin was significantly higher in F. This finding further provides evidence for the temperature-driven control over Rmin. Meanwhile, the higher SOC in L42 compared to NL was also the primary reason for its significantly higher Rmin, which did not fully support the second hypothesis. BD is another important factor affecting Rmin in forest ecosystems [5]. Lower BD typically corresponds to well-aggregated soil, which could create favorable conditions for N mineralization. Therefore, the higher soil BD in NL compared to L42 resulted in the lower soil Rmin. Simultaneously, soil labile C and N pools could provide available substrates and energy for microbial activity, thereby stimulating microbial processes and influencing Rmin [31]. Our study suggested that soil MBC and MBN concentrations in L42 were significantly higher than in NL, which could also lead to the higher Rmin.

Rmin was dominated by Rnit in our study, which is similar to other observations in temperate climates [32,33,34]. However, our results are contrary to the findings in cold temperate regions [35]. Compared to ammonifiers, previous studies have shown that nitrifiers perform best in higher temperatures [36]. In our study, the relative ST (more than 15 °C) induced a higher Rnit [37]. Soil Rnit, Ramm, and Rmin also showed significant seasonal dynamics in the present study (Figure 1). Soil Rmin was lowest in July and August, even producing negative values, reflecting net immobilization driven by peak plant N uptake and high-temperature microbial inhibition. This phenomenon may be due to these two reasons. First, in July and August, plants need substantial amounts of inorganic N for growth, which may induce higher inorganic N uptake than production by mineralization, and ultimately induce a lower Rmin. Second, the higher temperatures in this period might also inhibit microbial activity, thereby decreasing Rmin [38]. The higher Rmin in May and September might be due to the relatively lower temperatures and plants’ uptake of inorganic N, leading to the highest Rmin values.

### 3.2. Effects of Afforestation on Microbial Community

In our study, *Acidobacteria*, *Pseudomonadota*, *Actinobacteria*, *Chloroflexi*, and *Verrucomicrobiota* were the dominant bacterial phyla, while *Ascomycota*, *Basidiomycota*, and *Mucoromycota* were the dominant fungal phyla during afforestation. These findings suggest that afforestation did not alter the dominant microbial composition, which agrees with previous studies [39]. This is because all afforestation stages shared the same land-use history. Land-use history within the same region has a lasting effect on soil microbial community structure [39]. However, the relative abundance of dominant soil microbial communities differed significantly during afforestation. For soil bacterial communities, the relative abundance of *Acidobacteria* decreased during afforestation, while the relative abundance of *Pseudomonadota* peaked at the L42 stage in our study (Figure 6). Studies have shown that soil *Proteobacteria* are typical *r*-strategist bacteria, characterized by fast growth and dependence on nutrient-rich environments for metabolic activities. In contrast, *Acidobacteria* and *Actinobacteria* exhibit *K*-strategist ecological traits, preferring to colonize and proliferate in nutrient-poor habitats. With afforestation, the soil bacterial community composition gradually transitioned from *Acidobacteria*-dominant to *Pseudomonadota*-dominant, reflecting an ecological strategy shift from oligotrophic to copiotrophic groups, driven by increasing soil nutrient availability in our study (Figure 2). This finding is similar to the study conducted by Ren et al. [18], which also explained the higher relative abundance of *Pseudomonadota* in L42 compared to NL. The relative abundance of the fungal phylum *Ascomycota* increased with afforestation in this study (*p* < 0.05), which could be because *Ascomycota* are widely recognized as copiotrophic microorganisms and positively correlated with soil nutrient availability [40,41]. This further explains the higher relative abundance of *Ascomycota* in L42 compared to NL. Additionally, during afforestation, there were no significant differences in soil bacterial diversity, whereas fungal diversity exhibited a decreasing trend (F > L21 > L42) (Table 1). Compared to bacteria, the soil fungi community showed lower resistance and stability to land use change owing to their distinct ecological strategies [42]. Therefore, soil fungal diversity was more sensitive to afforestation than bacterial diversity in this study. However, compared to F, forest land with higher diversity and stability could provide more substrate for microbes, but did not result in higher soil fungal diversity. This result also agrees with previous studies [26]. This is because tillage may disrupt the existing microbial community structure while increasing the likelihood of successful microbial immigration [43]. Simultaneously, tillage may generally benefit communities by broadening the range of environmental conditions, preventing the dominance of certain taxa, and allowing more species to persist through reduced competitive exclusion, thus exerting positive effects on communities [26]. However, long-term soil modification by tree roots in planted forests homogenizes soil physical and chemical indicators such as bulk density, porosity, moisture and nutrients throughout the forest area. The dramatic reduction in microhabitat types consequently makes it impossible to support the survival of diverse fungal communities [44]. Meanwhile, there is a lower disturbance in natural forests than in plantations [45]. Microhabitat types in NL remain stable, consequently supporting the survival of diverse fungal communities; thus, fungal diversity in NL soils was significantly higher than in L42 in our study.

Microbial biomass also differed significantly during afforestation (Figure 6). Both MBC and MBN showed increasing trends with afforestation, which could be attributed to two reasons. First, SWC is a vital factor influencing soil microbial growth, and higher SWC could promote microbial biomass accumulation [46]. In this study, SWC showed an increasing trend with afforestation, which resulted in the highest microbial biomass occurring in L42. Secondly, soils with higher substrate availability usually mean higher microbial biomass [5,7]. In this study, the soil substrate availability showed an increasing trend during afforestation (Figure 1). Therefore, MBC and MBN contents followed the order L42 > L21 > F. Additionally, intensive anthropogenic disturbances such as tillage and fertilization in F could also result in significantly higher MBC and MBN contents in L21 than in F in our study [7]. The significantly higher SWC, TN, and SOC concentrations in L42 compared to NL could also lead to higher MBC and MBN concentrations.

### 3.3. Regulatory Mechanisms of Afforestation on Soil Nitrogen Mineralization

The variation in soil Rmin during afforestation was attributable to soil properties and microbial factors in our study (Figure 8). The RDA showed that there was a significant negative correlation between Rmin and pH (*p* < 0.01). Generally, there is a higher substrate supply (e.g., NH_4_^+^) for nitrification in lower-pH soils [47], which ultimately could enhance Rmin. Thus, low soil pH conditions could result in higher soil Rmin in our study, consistent with Hu et al. [13] in temperate meadow steppes. Additionally, Rmin was significantly positively related to the TN and SOC (*p* < 0.05). This is because abundant substrates can strongly stimulate activity and ultimately promote soil Rmin [5,30]. Although the soil C:N ratio plays a key role in regulating microbial activity and Rmin, no significant correlation was found between soil C:N ratio and Rmin in this study (*p* > 0.05). Rmin was also significantly correlated with soil bacterial and fungal communities. Previous studies have suggested that *Actinobacteria* are a prominent factor in regulating soil nitrogen cycling by producing extracellular enzymes and forming symbiosis with plants [48]. Meanwhile, as the main decomposer in the fungal community, the abundance of *Ascomycota* also has a positive relationship with Rmin [23,41]. Our study found that the relative abundances of *Actinobacteria* and *Ascomycota* gradually increased during afforestation, which could also explain the increasing trend in soil Rmin with afforestation.

The SEM results revealed that afforestation influenced soil Rmin through its effects on ST and NH_4_^+^ content. ST had a significantly direct effect on Rmin, which is consistent with previous studies [49]. ST is directly linked to soil Rmin by stimulating microbial activity, thereby enhancing soil Rmin [29]. NH_4_^+^ directly affected Rmin because nitrification serves as the primary component of Rmin in our study [50]. Meanwhile, soil NH_4_^+^ mainly acts as the substrate for microbial mineralization [51], and thus also indirectly influenced Rmin by affecting microbial communities in our study.

### 3.4. Implications

Our study suggested that afforestation could alter soil inorganic N pools and N transformation processes in Northeast China, and there were positive effects of ecological restoration through larch planting on soil N availability. However, the present study still has its limitations. Our study included only 21- and 42-year-old plantations and might not have captured all of the variations in the full dynamics of soil Rmin following afforestation, particularly for the initial restoration phase and long-term stable stage. Therefore, it is necessary to comprehensively consider more restoration stages in future forest management research, beyond the three restoration years examined in this study.

## 4. Materials and Methods

### 4.1. Study Region

The study was conducted in the Xiaoling of Heilongjiang Province in Northeast China (127°05′–127°34′ E, 45°23′–45°52′ N), which belongs to the Zhangguangcai vein. With an average altitude of 570 m, the climate in this area is characterized by a temperate continental monsoon climate, with an average annual temperature of 2.0 °C. The average annual precipitation is 550–700 mm, with 85% of it occurring between May and September and the average annual evaporation is 930 mm [52]. The frost-free period is approximately 130 d. The soils are grouped as Alfisols (Eutroboralfs) according to the United States Soil Taxonomy.

### 4.2. Soil Sampling and Analyses

In May 2024, we selected a typical afforestation restoration sequence, including a farmland (cultivated maize field, F), 21-year-old larch plantation (L21), and 42-year-old larch plantation (L42), with natural larch forest (NL) selected as a control. A total of twelve 20 m × 20 m plots were established, with three replicates at each stage and spaced at least 50 m apart. The basic information at each stage along the afforestation sequence was presented in Table 2.

Using the incubation method, soil Net Rmin was measured in situ with polyvinyl chloride plastic (PVC) cores [35]. Specifically, in each plot, three replicate soil cores (20 cm length, 5 cm diameter) were incubated randomly in the field for one month. At each time, pairs of PVC tubes were initially hammered 20 cm into the soil for soil sampling at each sampling point. One of the tubes was immediately brought to the laboratory to determine the initial inorganic N (NH_4_^+^-N and NO_3_^−^-N) concentrations. Another tube was sealed with permeable plastic film on the top and gauze under the bottom to prevent water penetration and allow gas exchange, and then placed into the soil. The tubes were incubated for approximately 30 d in each stage. At the end of incubation, the incubated samples were taken out and sent to the laboratory as the final samples. Meanwhile, another three soil samples at depths of 0–10 cm and 10–20 cm were also collected at each plot using a 5 cm diameter stainless-steel corer. And soil samples from the same layer were mixed into a composite sample, removing the litter, coarse debris, and stones by hand. All soil samples were transformed to the laboratory and sieved through a 2 mm mesh, and then stored at 4 °C for soil physicochemical analysis. At each sampling time, soil temperature (ST) was measured in each plot using a portable thermometer with a thermocouple probe (Delta TRAK, Pleasanton, CA, USA).

Additionally, three other soil samples were also collected from each stage to determine soil microbial community characteristics in the middle of July 2024. Before sampling, the surface litter and debris were cleared and the soil cores were collected. Similarly, in each plot, three soil samples were collected and mixed into a composite sample, and a total of 12 composite samples were obtained. All samples were stored at −80 °C. The specific method was presented in Supplementary Material S1.

### 4.3. Soil Analysis

Soil NH_4_^+^-N and NO_3_^−^-N concentration was determined by adopting the method of 1 mol·L^−1^ KCl solution extraction, and the filtrate was measured using an AA3 continuous flow auto-analyzer (AutoAnalyzer-AA3, Norderstedt, Germany). Soil total inorganic nitrogen content was the sum of the NH_4_^+^-N and NO_3_^−^-N contents. Soil water content (SWC) was determined gravimetrically by oven drying the soil samples at 65 °C until constant weight. Soil bulk density (BD) was determined using a circular stainless steel cutting ring. The soil pH was determined by a pH meter (FE20, Mettler Toledo, Shanghai, China) with a 1:2.5 mixture (soil:water ratio). Soil organic carbon (SOC) and total nitrogen (TN) were determined using the MultiC/N 3000 analyzer (Anailtik Jena AG, Jena, Germany) with the HT1500 Solids Module and the semi-micro Kjeldahl method. Soil microbial biomass carbon (MBC) and nitrogen (MBN) were determined using the fumigation method [40].

### 4.4. Data Analysis

The differences in soil Rmin, soil properties, and microbial community composition across different stages of afforestation were examined using One-way ANOVA followed by Duncan’s test, after which the data were tested for normality and homoscedasticity. The correlations between soil Rmin and its related factors were examined by Pearson correlation analysis, with the significance level at *p* < 0.05. All statistical analyses were performed using SPSS software version 21.0 (SPSS Inc., Chicago, IL, USA). The relationships among soil Rmin, properties, and microbial community characteristics were determined by redundancy analysis (RDA) in Canoco 4.5 [53]. Principal coordinate analysis (PCoA) was performed to visualize the differences in soil bacterial and fungal communities among afforestation stages. All graphs in this study were plotted using Origin 2024 software (OriginLab Corporation, Northampton, MA, USA).

### 4.5. Structural Equation Modeling

A structural equation model (SEM) was constructed to comprehensively explore the hypothetical pathway through which afforestation affects soil Rmin by potentially regulating soil nitrogen availability and microbial community structure using IBM Amos 20.0. The direct and indirect pathways of afforestation affecting soil Rmin were quantified. The P and correlation coefficients (r values) from the Mantel test between any two parameters. The goodness-of-fit statistics were determined using the Chi-square test (*p* > 0.05), goodness-of-fit index (GFI > 0.90), and root mean square error approximation (RMSEA < 0.05).

## 5. Conclusions

This study employed an in situ field experiment to investigate the effects of larch (*Larix gmelinii*) afforestation on soil Rmin in Northeast China. Soil Rmin initially decreased, then increased with afforestation. Compared with NL, soil Rmin in L42 was significantly higher, indicating that mature plantations enhance N mineralization beyond natural forest levels. Additionally, the soil microbial community gradually transitioned from oligotrophic to copiotrophic groups during afforestation, reflecting a shift in microbial life-history strategies that accelerate organic matter decomposition and nutrient release. Afforestation affects soil Rmin through alterations in ST and NH_4_^+^ content. These changes suggest that afforestation progressively enhances the soil nutrient supply capacity, favoring long-term forest productivity and carbon–nitrogen retention. In conclusion, this study reveals the interactions among afforestation, microbes and nitrogen mineralization, and highlights the associated soil and ecosystem processes. These findings provide key information on the carbon-nitrogen balance of afforestation in Northeast China.

## Figures and Tables

**Table 1:** The alpha diversity of bacteria and fungi communities at four stages.

Microbial Communities	Alpha Diversity Index	F	L21	L42	NL
Bacteria	Chao1	3896.99 ± 139.72 b	3600.28 ± 248.62 b	3851.68 ± 140.03 b	4921.80 ± 310.47 a
	ACE	3980.71 ± 146.09 b	3661.10 ± 210.03 b	3906.84 ± 175.16 b	5012.52 ± 352.38 a
	Shannon	7.06 ± 0.09 a	7.01 ± 0.15 a	7.08 ± 0.06 a	7.18 ± 0.03 a
	Simpson	1.00 ± 0.00 a	1.00 ± 0.00 a	1.00 ± 0.00 a	1.00 ± 0.00 a
Fungus	Chao1	906.96 ± 12.30 a	912.16 ± 10.97 a	686.83 ± 148.66 b	426.50 ± 18.69 c
	ACE	908.06 ± 24.61 a	891.57 ± 19.29 a	684.36 ± 153.66 b	417.54 ± 1.72 c
	Shannon	4.12 ± 0.32 a	4.02 ± 0.04 ab	3.77 ± 0.13 b	4.37 ± 0.04 a
	Simpson	0.91 ± 0.02 b	0.93 ± 0.00 b	0.90 ± 0.00 c	0.97 ± 0.00 a

Note: Lowercase letters indicate significant differences between afforestation stages in each index (*p* < 0.05).

**Table 2:** Basic overview of the sample plots in the four stages of returning afforestation.

Stage	F	L21	L42	NL
Afforestation duration/a	0	21	42	-
Altitude/m	240.2 ± 2.1	243.5 ± 1.8	282.1 ± 2.5	313 ± 4
Slope	<15	<15	<15	<15
DBH/cm	-	12.06 ± 0.45	14.9 ± 1.25	14.89 ± 4.40
Height/m	-	5.7 ± 0.26	9.43 ± 0.4	11.42 ± 1.05
Forest density/(trees⋅ha^−1^)	-	3000	2400	1400
BD/g cm^−3^	0.88 ± 0.06 a	0.56 ± 0.03 c	0.69 ± 0.06 b	0.88 ± 0.12 a
TN/g kg^−1^	1.6 ± 0.1 b	1.6 ± 0.3 b	4.2 ± 0.2 a	1.2 ± 0.1 c
SOC/g kg^−1^	25.55 ± 1.18 b	26.7 ± 0.26 b	65.6 ± 3.25 a	23.91 ± 0.86 b
C:N	15.75 ± 1.24 b	17.11 ± 3.29 ab	15.83 ± 1.22 b	19.82 ± 1.37 a
pH	5.67 ± 0.02 c	5.78 ± 0.06 b	5.56 ± 0.02 d	5.98 ± 0.02 a

Note: Lowercase letters indicate significant differences between afforestation stages in each index (*p* < 0.05).

## Data Availability

The sequencing data of soil bacteria and fungi have been deposited in the National Center for Biotechnology Information (NCBI) under accession numbers PRJNA1055189.

## Supplementary Materials

The following supporting information can be downloaded at: https://www.mdpi.com/article/10.3390/plants15121892/s1.
