# Supplementary material for: Soil Nutrients, pH and Microorganisms Modulate Nitrogen Mineralization Dynamics Following Afforestation in Northeastern China
Source: Plants (Basel). 2026 Jun 18;15(12):1892. doi: 10.3390/plants15121892 (PMC13306838; doi:10.3390/plants15121892)
Supplement: Supplementary file 1 [file plants-15-01892-s001.zip › plants-4367381-supplementary.pdf]

Supplementary File(s)

Soil Nutrients, pH and Microorganisms Modulate Nitrogen Mineralization Dynamics Following Afforestation in Northeastern China

Lei Guo [1,2], Xu Cao [1,2], Ruihan Xiao [1,2,3], Kexin Tong [1,2], Tao Liu [1,2], Minghan Lang [4] and Beixing Duan [1,2,3,*]

1 School of Hydraulic and Electric Power, Heilongjiang University, Harbin 150080, China; guolei@s.hlju.edu.cn (L.G.); coxi@s.hlju.edu.cn (X.C.); xiaoruihan@hlju.edu.cn (R.X.); 2022074@hlju.edu.cn (K.T.); 2002219@hlju.edu.cn (T.L.)

2 International Joint Laboratory of Hydrology and Hydraulic Engineering in Cold Regions of Heilongjiang Province, Harbin 150080, China

3 Post-Doctoral Mobile Research Station of Ecology, Heilongjiang University, Harbin 150080, China

4 Liaoning Zhanggutai Desert Ecosystem Research Station, Liaoning Institute of Sandy Land Control and Utilization, Fuxin 123000, China; 18804502009@163.com

* Correspondence: duanbx@hlju.edu.cn; Tel.: +86-182-4605-7664

S1 Additional Materials and Methods

S1.1 Microbial sequencing

DNA was extracted from the soil using a PowerSoil® DNA Isolation Kit (MoBio Inc., Carlsbad, USA). Agarose gel electrophoresis and a Nanodrop1000 Spectrophotometer (Thermo Fisher Scientific, USA) were used to visualize and quantify the extracted DNA. The V3–V4 region of the 16S rRNA gene was amplified with primer pairs 338F/806R (5′-ACTCCTACGGGAGGCAGCAG-3’/5′-GGACTACHVGGGTWTCTAAT- 3′). A PCR was run with the following parameters: one cycle at 95°C for 5 min; 27 cycles of 94 °C for 30 s, 55 °C for 30 s, and 72 °C for 45 s; and a final cycle of 72 °C for 10 min. The PCR products were subsequently purified and sequenced on the Illumina Hiseq 2500 platform (Magigene Co., Ltd, Guangzhou, China). Sequence analysis was conducted using the QIIME 1.6.0 pipeline software. The sequences were assigned to operational taxonomic units (OTUs) with Uparse software at a similarity of 97%. The raw sequences retrieved in

this study have been deposited in the National Center for Biotechnology Information (NCBI) under accession numbers PRJNA797560.

S1.2 Soil temperature

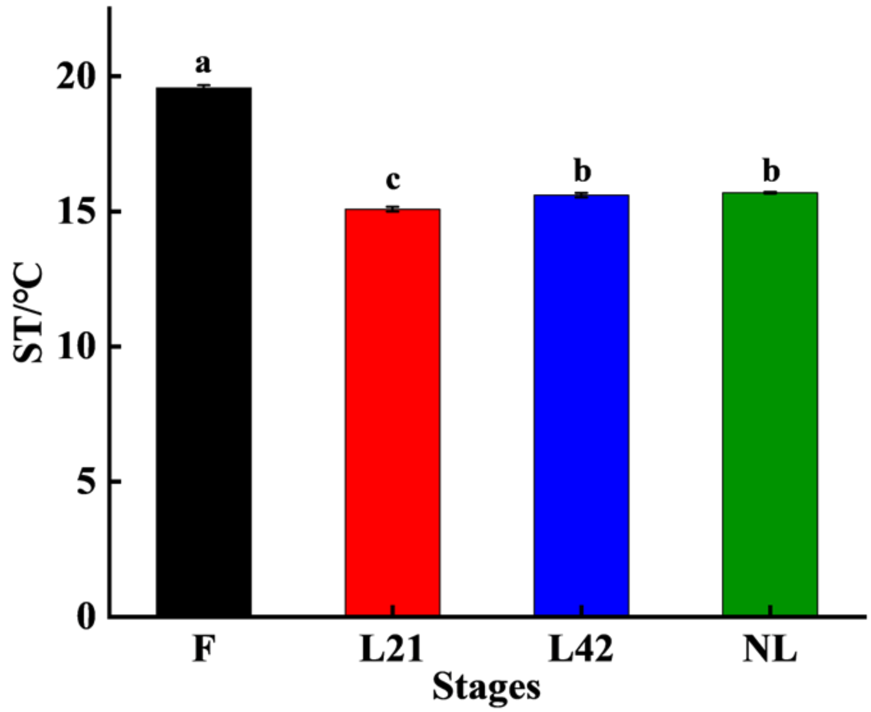

Figure S1. Dynamics of soil temperature following afforestation. Notes: Lowercase letters indicate significant differences among the different stages.
